# Nursing Workload in Patients With Myocardial Ischemia After Non-cardiac Surgery

**DOI:** 10.7759/cureus.30737

**Published:** 2022-10-26

**Authors:** Panagiota Manthou, Georgios Lioliousis, Ioustini Pietri, Panagiotis Vasileiou, Dimitris Dainavas, Georgios Fildisis

**Affiliations:** 1 Nursing, National and Kapodistrian University of Athens, Athens, GRC; 2 Intensive Care Unit, First Department of Respiratory Medicine, Thoracic Diseases General Hospital Sotiria, National and Kapodistrian University of Athens, Athens, GRC; 3 Nursing, General Oncology Hospital of Kifissia, Athens, GRC; 4 Laboratory of Histology & Embryology, National and Kapodistrian University of Athens School of Medicine, Athens, GRC; 5 Emergency Department, Thoracic Diseases General Hospital Sotiria, National and Kapodistrian University of Athens, Athens, GRC; 6 Intensive Care Unit, General Oncology Hospital of Kifisia, Athens, GRC; 7 Nursing School, National and Kapodistrian University of Athens, Athens, GRC

**Keywords:** non cardiac surgery, myocardial ischemia, icu, nursing activity score (nas), nursing workload (nwl)

## Abstract

Introduction: Nursing workload (NWL) in the intensive care unit (ICU) is an essential parameter of patient safety. However, little attention has been dedicated to measuring NWL in ICU about patients surgically treated with myocardial ischemia (MI).

Methods: The objectives of this study are to describe and examine the NWL by applying the Nursing Activities Score in patients who underwent non-cardiac surgery and developed MI in the ICU. The statistical significance was set at 0.05. The statistical program SPSS 22.0 was used for the analysis.

Results: The mean age was 69.1 years, whereas 32.4% of the patients had MI. Hypertension, diabetes mellitus, and dyslipidemia were the main comorbidities. On the first day in ICU, the NWL was similar in all patients (p = 0.947). In the following days, the NWL was significantly higher in patients with MI (p < 0.001). The NWL was considerably higher in patients with MI who died.

Conclusions: The present results are essential for planning and using nursing resources according to the care needs of postoperative patients with MI.

## Introduction

Cardiovascular complications following non-cardiac surgery are associated with high morbidity and mortality. Worldwide, more than 200 million adults undergo some form of non-cardiac surgery each year [1]. Patients with comorbidities such as dyslipidemia, hypertension, and diabetes mellitus have higher rates of perioperative or postoperative myocardial ischemia (MI). The mortality for patients who may need care in the intensive care unit (ICU) is higher [2,3]. It is essential to improve the perioperative and postoperative outcomes of high-risk patients for MI through preoperative identification of risk factors and prevention to reduce the prevalence and severity of postoperative myocardial ischemia (POMI) [4]. However, the lack of experienced nursing staff in the ICU remains a significant problem. The literature review proved that patients with various health problems admitted to ICUs have negatively correlated mortality with the duration and cost of hospitalization [5,6]. 

The Nursing Activities Score (NAS) is one of the tools for measuring nursing workload (NWL) in ICUs. It consists of 23 items of basic nursing activities, each representing the time a nurse has to provide care to a seriously ill patient during a 24-hour hospitalization in the ICU [7]. The sum of the 23 NAS objects ranges from 0 to 177%. Each grade of the grading system represents the average time required for each nursing activity within 24 hours, corresponding to 14.4 minutes of nursing care. In addition, 100 units of total NAS load represent 100% of a nurse's nursing time per working hour. The scale is evaluated based on the real time that each nursing intervention lasts, regardless of the severity of the disease. Its Greek translation has been weighted in cardiology units, with a satisfactory internal consistency reliability factor of Cronbach’s α equal to 0.65 [8]. Despite the importance of measuring NWL using NAS, no studies correlate the NWL in patients who develop POMI. 

## Materials and methods

The data for this study were obtained from a database containing information on patients who underwent non-cardiac surgery at a general hospital in Athens. Patient anonymity was maintained. After approval from the ethics committee of the Department of Nursing of the National Kapodistrian University of Athens (20/06/2018-266) and after written informed consent, data from 105 consecutive patients admitted to ICU between June 2018 and June 2021 after non-cardiac surgery were collected. Inclusion criteria were patients of both sexes, aged ≥18 and <85 years, who had been admitted for non-cardiac surgical procedures and needed to stay in the ICU for more than 24 hours. Exclusion criteria were age <18 years because comorbidities such as dyslipidemia, hypertension, and diabetes mellitus are less common, length of ICU stay <24 hours, and ICU readmissions. Ischemia monitoring was performed in each patient only after the first surgery. Written informed consent was obtained preoperatively from all eligible patients or their proxies. Patients (1) who refused consent, (2) who were unable to give consent, (3) whose consent could not be obtained for logistical/emergency reasons, or (4) who were diagnosed with MI before surgery were excluded. The ICU had 12 beds.

The nursing team worked per 8-hour shift and the strength of the nursing staff in the ICU was 25 nurses and two nursing assistants. The staffing followed the nurse-to-patient ratio of 1: 3. Normally, better ratios between nurses and patients are needed so as to provide better care. The reason for this ratio in this research is that there was a lack of health-care staff. Information collected from medical records included data from the first 24 hours of ICU admission, such as age, sex, predicted surgical mortality, comorbidities, cardiac history, surgery history and type of surgery, and the administered vasoactive drugs. Clinical data such as diuresis, blood gas measurements, and ejection fraction were recorded. Patient data at ICU discharge, length of ICU stay (days), and duration of hospitalization were also collected. For each patient, the NAS was calculated for the first three days of hospitalization in the ICU. Since all patients (n = 105) were hospitalized for at least three days in the ICU, a total of 315 measurements were done.

Statistical analyses

Mean values, standard deviations (SDs), median, and interquartile range were used to describe the quantitative variables. Absolute (N) and relative (%) frequencies were used to describe the qualitative variables. Pearson’s χ^2^ test or Fisher’s exact test was used to compare the ratios, where necessary. The Student's t-test or the Mann-Whitney non-parametric criterion was used to compare the quantitative variables between the two groups. The analysis of variance for repeated measures (ANOVA) was used to check for differences in the NWL between the groups and time. Also, with the above method, it was assessed whether the degree of change in the load time was different between the two groups. The significance levels are bilateral and the statistical significance was set at 0.05. The statistical program SPSS 22.0 (IBM Corp., Armonk, NY) was used for the analysis. When the normality assumption for the comparison of means between two groups was met, the student’s t-test was used. 

## Results

Demographic characteristics of a study sample

Failure of weaning and hemodynamic instability were the main factors for patients admitted from the post-anesthetic unit to the ICU. Most of them were men (53.3%) and had a mean age of 69.1 years (SD = 11.3). During the first three days of hospitalization in the ICU, 32.4% developed MI. The MI percentages did not differ significantly depending on gender, blood type, rhesus, or history of allergy. However, patients with MI were significantly older. The demographic data of patients are presented in Table 1.

**Table 1:** Demographic data MI, myocardial ischemia; SD, standard deviation. +Pearson’s x 2 test; ++Fisher’s exact test; ‡Student’s t-test.

		Total sample (N = 105, 100%)	Without MI (N = 71, 67.6%)	With MI (N = 34, 32.4%)	
		Ν (%)	Ν (%)	Ν (%)	p-Value
Sex	Men	56 (53.3)	36 (50.7)	20 (58.8)	0.435+
	Women	49 (46.7)	35 (49.3)	14 (41.2)	
Mean age (SD)		69.1 (11.3)	67.5 (11.8)	72.4 (9.7)	0.034‡
Blood type	A	40 (38.5)	23 (32.4)	17 (51.5)	0.229++
	B	18 (17.3)	12 (16.9)	6 (18.2)	
	AB	5 (4.8)	4 (5.6)	1 (3)	
	O	41 (39.4)	32 (45.1)	9 (27.3)	
Allergy	No	84 (80.8)	55 (65.5)	29 (34.5)	0.414
	Yes	20 (19.2)	15 (75)	5 (25)	

The majority of surgeries featured were gastroenterological and thoracic surgeries. Also, the descriptive data show that 84.8% of the patients had some co-existing disease. More specifically, 54.3% suffered from hypertension, 23.8% from dyslipidemia, 21% from chronic obstructive pulmonary disease, 24.8% from diabetes, and 75.2% from another disease (Table 2). MI rates were significantly higher in patients with at least one comorbidity upon admission to the hospital (p < 0.015).

**Table 2:** Comorbidities before surgery MI, myocardial ischemia; COPD, chronic obstructive pulmonary disease. +Pearson’s x^2^ test.

		Total sample (N = 105, 100%)	Without MI (N = 71, 67.6%)	With MI (N = 34, 32.4%)	p-Value+
		Ν (%)	Ν (%)	Ν (%)	
Comorbidities	No	16 (15.2)	15 (93.8)	1 (6.3)	0.015
	Yes	89 (84.8)	56 (62.9)	33 (37.1)	
Hypertension	No	48 (45.7)	40 (83.3)	8 (16.7)	0.002
	Yes	57 (54.3)	31 (54.4)	26 (45.6)	
Dyslipidemia	No	80 (76.2)	60 (75)	20 (25)	0.004
	Yes	25 (23.8)	11 (44)	14 (56)	
COPD	No	83 (79)	60 (72.3)	23 (27.7)	0.047
	Yes	22 (21)	11 (50)	11 (50)	
Diabetes mellitus	No	79 (75.2)	58 (73.4)	21 (26.6)	0.027
	Yes	26 (24.8)	13 (50)	13 (50)	
Other	No	26 (24.8)	22 (84.6)	4 (15.4)	0.033
	Yes	79 (75.2)	49 (62)	30 (38)	

All patients' median hospitalization duration was 25 days (range: 15-35). Patients who had postoperative MI had a longer mean hospital stay with a mean duration of 32 days (median range: 25-45) compared with the control group who had a median duration of 18 days (median range: 14-30) (p < 0.001 ). The length of stay in the ICU, sedation, and mechanical respiration support were significantly higher in patients with MI (p < 0.001). Mortality in these patients was 72% compared to the control group, where the mortality was about 21% (p < 0.001) (Table 3). The multifactorial analysis showed that patients with MI were 4.34 times more likely to die than patients without MI.

**Table 3:** Clinical data regarding hospitalization in ICU MI, myocardial ischemia; SD, standard deviation; IQR, interquartile range; ICU, intensive care unit.

		Total sample (N = 105, 100%)	Without MI (N = 71, 67.6%)	With MI (N = 34, 32.4%)	p-Value
		Ν (%)	Ν (%)	Ν (%)	
Length of stay (days), SD (IQR)		27.9 (18.4), 25 (15-35)	24.4 (17.6), 18 (14-30)	35.3 (18), 32.5 (25-45)	0.001
Length of stay in ICU (days), SD (IQR)		13.8 (14.9), 9 (4-17)	10.5 (13.3), 5 (3-11)	20.7 (15.9), 17.5 (10-25)	<0.001
Length of stay in sedation (days), SD (IQR)		9.6 (13.7), 4.5 (1-12)	6 (10.7), 2 (1-6)	17.1 (16.1), 11.5 (7-22)	<0.001
Length of stay in mechanical ventilation (days), SD (IQR)		9.9 (13.7) 5 (1.5-12.5)	6.6 (11.1), 2.5 (1-7)	16.8 (16.1), 12.3 (7-21)	<0.001
Outcome	Exit of ICU	83 (79)	65 (78.3)	18 (21.7)	<0.001
	Death	22 (21)	6 (27.3)	16 (72.7)	

Comparison of NAS with the occurrence of MI

At 24 hours, NWL was similar in both groups. On the second and third days, the NWL was significantly higher in patients with MI (p < 0.001). More analytically, comparing per day, it was found that in patients without MI, the NWL was similar in the first days (p = 0.756), while on the third day, it decreased significantly (p < 0.001). In contrast, in patients with MI, the NWL increased significantly from the first to second day (p < 0.001), from the second to third (p = 0.021), but also overall from the first to the third day (p < 0.001) (Table 4).

**Table 4:** Quantitative measurement of nursing workload MI, myocardial ischemia; SD, standard deviation.

	Nursing Workload			
	First day	Second day	Third day	Overall change between first and third days
Groups	Mean (SD)	Mean (SD)	Mean (SD)	Mean (SD)
Without MI	58.84 (19.27)	56.94 (17.52)	53.38 (18.16)	-5.46 (13.56)
With MI	60.41 (18.31)	72.76 (16.61)	77.3 (15.48)	16.89 (16.85)

**Figure 1:**
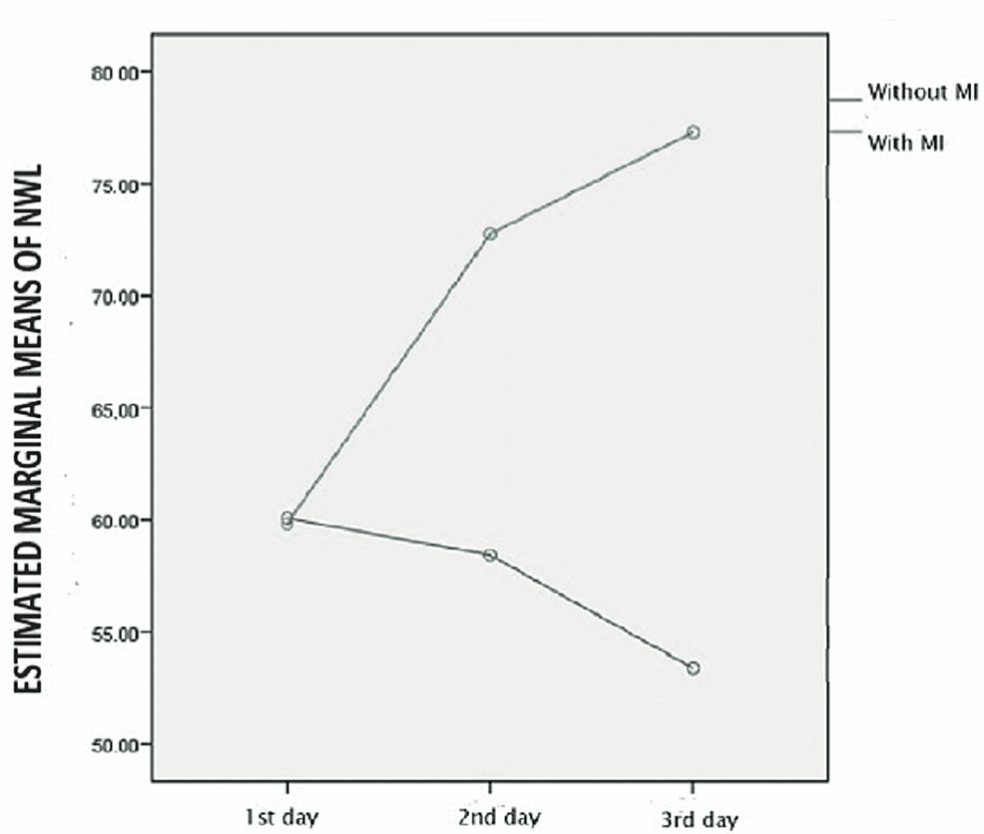
Rate of change of NWL between the two groups NWL, nursing workload; MI, myocardial ischemia.

The degree of change differed significantly between the two groups (p < 0.001), as shown in the following graph (Figure 1). 

## Discussion

Studies on NWL are based primarily on hours of nursing care. The participants in this study were old adults with high severity of illness; most patients had undergone primary surgical treatment, were transferred from the operating room, and had a median ICU stay of 13 days. Also, the increased workload levels for each patient during the first three days of hospitalization in the ICU were associated with a statistically significant increase in the total length of hospitalization. The NWL at ICUs was high since 5.82 hours of direct nursing care were provided per 8-hour shift [NAS(8 h) = 100%]. This result shows the mean number of hours (5.81 hours) a nurse spends in the direct care of only one patient during an 8-hour shift. During the remaining 2.18 hours, a nurse could help another professional care for a different patient.

From the international literature review, no studies correlate the effect of NWL during the treatment of patients who have undergone major surgery and developed postoperative MI. In the present study, the outcome was associated with the NWL in patients with MI, and it was found that 1.4 higher NAS scores were recorded on the second day and 1.6 NAS scores on the third day in the patients who eventually died. Also, no study was observed correlating the length of hospital stay with the mortality of patients who have undergone major surgery. However, several studies have crosslinked the length of hospital stay in the ICU with patient mortality, concluding that prolonged hospital stay in the postoperative ICU is associated with increased mortality [9,10]. A study of 735 control patients who underwent coronary artery bypass graft surgery concluded that patients who died were hospitalized in the ICU for longer than the others [11]. The present study revealed a correlation between nursing care and length of stay in the ICU. Prolonged hospitalization in the ICU also includes a series of side effects such as nosocomial infections and nursing staff burnout. 

The highest NWL with mean NAS (SD) = 77.3 (15.48) in patients with MI appeared to increase gradually, mainly on the third day of stay in the ICU, which was associated with MI between the second and third day. Thus, these patients required more intensive care. The highest NWL with mean NAS (SD) = 58.84 (19.27) was recorded in the control group on the first postoperative day. This result is in line with the average value of the NAS scale in postoperative patients treated in general ICUs. In addition, the need for intensive care to restore patient hemodynamic stability implies a more significant number of nursing interventions and, consequently, a greater workload. It is emphasized that, in the immediate postoperative period, patients with complications need closer monitoring of vital signs (especially respiratory parameters) [12-14]. 

The present study had no significant correlation of NWL with the patient's age. Similar findings are found in other studies in postoperative patients [15,16]. However, high-risk patients to develop MI had a greater possibility of prolonging the length of ICU stay and, therefore, higher NWL. That is why it is essential to develop a non-cost-effective protocol, easy to use and suitable for non-cardiac surgery patients. This will help the health-care staff recognize that patients are likely to develop MI and apply personalized care to reduce ICU days and staff workload [15]. 

In conclusion, the severity of illness and the presence of MI after non-cardiac surgery were the only factors associated with the NWL in the ICU. Despite the significant contributions, we acknowledge some limitations to our study, including that it was conducted with a convenience sample in the university ICU of a large general hospital in Athens. In addition, although NAS is a valid and reliable tool for measuring NWL, there is no internationally accepted way of calculating NWL. Also, in the present study, the NWL was calculated for each patient separately, without reference to the number of nursing staff required at each shift. Moreover, the number of studies on the use of the NAS in this category of patients is limited enough to allow comparisons. Still, the results suggest that personnel management should be based on specific characteristics of the patients at a high risk of developing postoperative complications such as MI.

## Conclusions

The appearance of MI also requires specialized postoperative intensive nursing care. However, the lack of nursing staff involves the identification of NWL for more efficient and effective management. The use and application of NAS as a tool for determining the NWL seem to be reliable for predicting the duration of hospitalization and the mortality of patients undergoing non-cardiac surgery and who are likely to develop MI.
